# Volumetric analysis of root coverage with porcine acellular dermal matrix: Case study

**DOI:** 10.1002/cap.70029

**Published:** 2026-02-19

**Authors:** Edgar Daniel Vargas‐Quiroga, Giovanna Fernanda Favero da Silva, Bruna Santos Honorio Tonin, Arthur Belém Novaes‐Jr, Marilia Bianchini Lemos Reis

**Affiliations:** ^1^ Department of Oral & Maxillofacial Surgery and Periodontology Ribeirão Preto School of Dentistry (FORP) University of São Paulo Ribeirão Preto Brazil; ^2^ Department of Restorative Dentistry Ribeirão Preto School of Dentistry (FORP) University of São Paulo Ribeirão Preto Brazil

**Keywords:** acellular dermal matrix, case reports, cone‐beam computed tomography, gingival recession, three‐dimensional image

## Abstract

**Background:**

Gingival recession is prevalent and often treated with coronally advanced flap plus connective tissue graft (CAF+CTG). Porcine acellular dermal matrix (PADM) offers a donor‐site‐free alternative. Contemporary digital imaging allows minimally invasive, three‐dimensional (3D) soft‐tissue assessment. This case report quantified 3D soft‐tissue changes after PADM‐based root coverage using an integrated digital workflow.

**Methods:**

A 38‐year‐old woman presenting with recession‐type 2 (RT2) defects on teeth #7, #6, and #5 underwent root coverage with an extended CAF in combination with a PADM. Three‐dimensional datasets were obtained at baseline, 6 months, and 12 months using intraoral scanning and cone‐beam computed tomography (CBCT). These were processed with image‐analysis software to assess volumetric and contour changes over time. Clinical parameters and patient‐reported satisfaction (visual analog scale, VAS) were also recorded.

**Results:**

Buccal soft‐tissue volume increased from 329.70 mm^3^ (baseline) to 454.81 mm^3^ (6 months), then to 345.25 mm^3^ (12 months), yielding a net gain of +4.72% versus baseline and a −24.09% reduction from the 6‐month peak. Color‐coded maps indicated predominant augmentation in the cervical third with contour stability at 12 months. VAS scores improved over time.

**Conclusions:**

PADM‐assisted root coverage increased gingival thickness and resulted in a stable buccal contour. 3D CT volumetric assessment seems to be a promising method for quantifying long‐term changes in regenerated tissues.

## Key points

- Porcine acellular dermal matrix‐assisted root coverage can increase gingival thickness and stabilize the buccal contour while avoiding donor‐site morbidity.
- A digital workflow enables precise, reproducible three‐dimensional volumetric assessment. Color‐coded deviation maps visualize spatial patterns of augmentation and remodeling beyond two‐dimensional metrics.
- In this case, volume rose from baseline to 6 months and partially contracted by 12 months, consistent with soft‐tissue maturation, with a net gain versus baseline.

## INTRODUCTION

Gingival recession (GR) is a common clinical condition characterized by the apical migration of the gingival margin relative to the cementoenamel junction, leading to root exposure.^1^, ^2^, ^3^ This condition may result in dentin hypersensitivity, compromised esthetics, and increased susceptibility to cervical abrasion and root caries, ultimately affecting both function and patient satisfaction.^1^, ^2^, ^3^

Epidemiological studies indicate that GR is highly prevalent worldwide. In South America, approximately 14% of adolescents and more than 90% of Chilean adults present with recession, predominantly Recession Type 2 and 3 (RT2/RT3) associated with periodontitis.^4^ Data from the U.S. National Health and Nutrition Examination Survey (NHANES 2009–2014) show that 33.8% of teeth in adults aged ≥30 years show GR ≥1 mm, more frequently in premolars and the mandibular arch; with only 0.85% classified as RT1, reinforcing the link with interproximal attachment loss.^5^ A four‐year cohort study conducted in Brazil reported a 35.9% incidence of new buccal GRs, particularly among individuals with advanced periodontitis.^6^

Among available surgical techniques, the coronally advanced flap combined with a connective tissue graft (CAF + CTG) remains the benchmark for achieving predictable root coverage, particularly in single‐site RT1/RT2 defects.^7^, ^8^, ^9^ However, CTG palatal harvesting involves donor‐site morbidity, increased surgical time, and limited graft availability.^10^ In this context, porcine acellular dermal matrix (PADM) has emerged as a promising alternative, offering advantages such as commercial availability, elimination of the donor site, and proven biocompatibility.^10^, ^11^, ^12^, ^13^

Recent advances in digital dentistry allow precise, minimally invasive assessment of soft and hard tissues. Integrated workflows combining intraoral scanning, cone‐beam computed tomography (CBCT), and other imaging modalities enable three‐dimensional (3D) volumetric analysis with high reproducibility.^14^, ^15^, ^16^, ^17^, ^18^, ^19^, ^20^, ^21^ These tools have identified associations between GR and buccal bone dehiscence, reduced keratinized tissue width, and a history of periodontal treatment.^22^ They also facilitate objective monitoring of graft integration and stability over time.^23^, ^24^, ^25^, ^26^, ^27^ This case report, prepared in accordance with the CARE guidelines,^28^ describes the clinical and volumetric outcomes of PADM‐assisted root coverage in a patient with multiple adjacent RT2 defects, using a fully digital workflow for 3D soft‐tissue evaluation at baseline, 6 months, and 12 months.

## MATERIALS AND METHODS

### Case study

**FIGURE 1:**
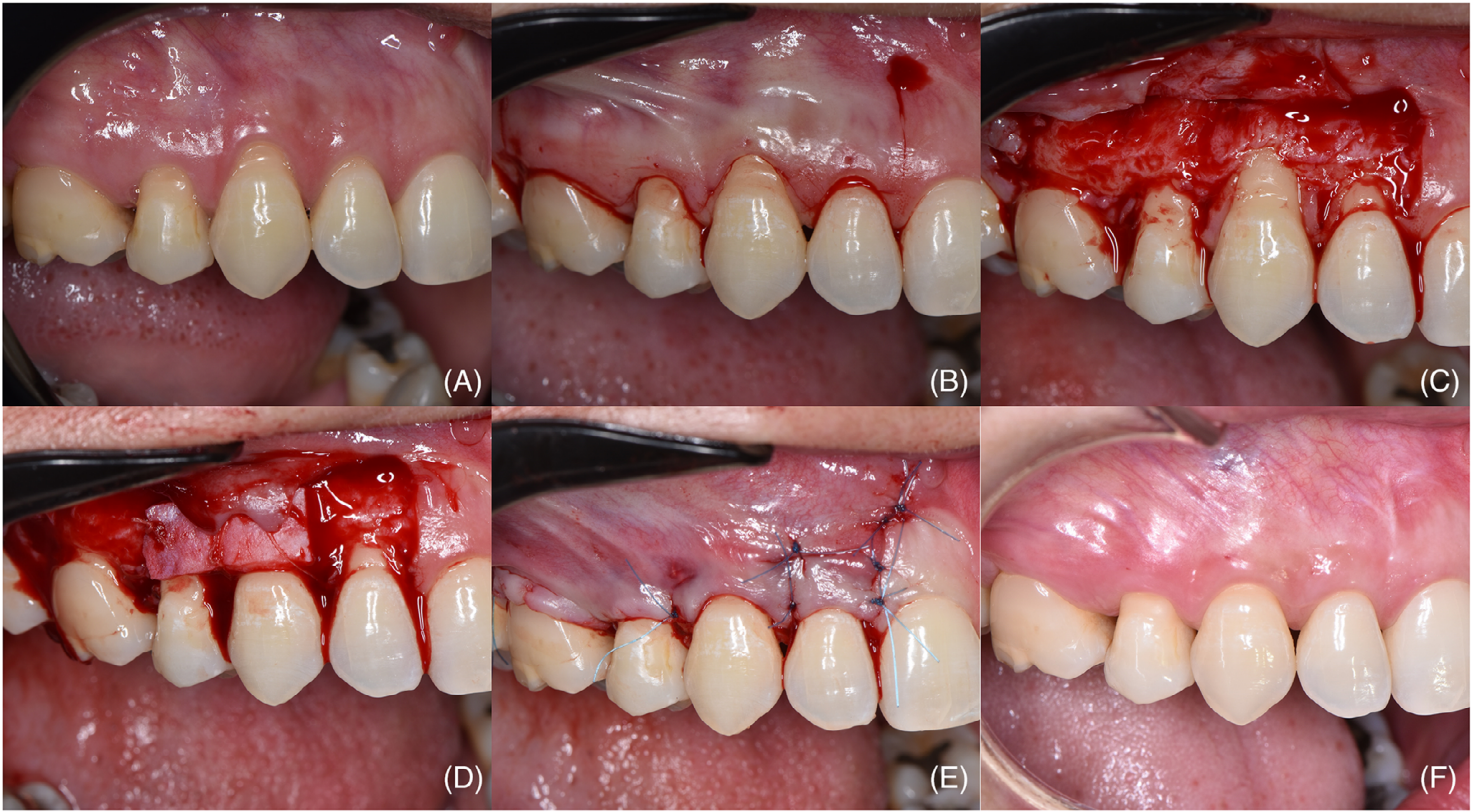
Clinical sequence of the root coverage procedure using a porcine acellular dermal matrix. (A) Initial clinical view. (B) Flap design with intrasulcular and vertical incisions. (C) Partial‐thickness flap elevation beyond the mucogingival junction and thorough root surface debridement. (D) Placement of the porcine acellular dermal matrix (PADM) trimmed and adapted 1 mm apical to the cementoenamel junction (CEJ). (E) Immediate postoperative view. (F) Clinical appearance at 12‐month follow‐up.

A 38‐year‐old female patient (ASA I), with no history of systemic disease, allergies, or smoking, sought treatment at the Department of Oral & Maxillofacial Surgery and Periodontology, School of Dentistry, University of São Paulo (FORP‐USP), Ribeirão Preto, Brazil, in April 2024. Patient presented with an esthetic concern related to multiple GRs in the maxillary right quadrant. Clinical examination revealed gingival health on a reduced periodontium in a non‐periodontitis patient, associated with GR^29^ classified as Cairo RT2,^30^ affecting the #7, #6, #5, and #3 (Figure 1A). The patient reported the use of a medium‐bristle toothbrush and having a recent history of orthodontic treatment. After obtaining informed consent, the patient underwent initial periodontal therapy, which consisted of individualized oral‐hygiene instruction, supragingival scaling, and root planing. This case report was approved by the FORP‐USP Research Ethics Committee (CAAE 90892125.4.0000.5419). The patient provided written informed consent for publication of de‐identified clinical images and data. Four weeks later, re‐evaluation confirmed the absence of bleeding on probing and stable periodontal conditions, indicating suitability for surgical intervention.

All clinical measurements were performed by the same experienced periodontist (Marilia Bianchini Lemos Reis),^31^ using a millimeter‐marked periodontal probe,* at baseline and at 12‐month follow‐up (Table 1). We recorded recession depth (RD), recession width (RW), keratinized tissue height (KTH), and gingival thickness (GT). Only teeth related to the patient's chief complaint (#7–#5) were included in the primary quantitative analysis. The first molar (#3) lay outside the esthetic field and was monitored clinically only.

**TABLE 1:** Clinical parameters measured at baseline, 6 months, and 12 months in the treated teeth.

	Baseline (mm)				6 months (mm)				ΔGT (6 months − baseline) (mm)^*^	12 months (mm)				ΔGT (12 months − baseline) (mm)^*^
Tooth	RD	RW	KTH	GT	RD	RW	KTH	GT	ΔGT^*^	RD	RW	KTH	GT	ΔGT^*^
#7	1	3	4	0.80	0	0	3	1.85	+1.05	0	0	4	1.65	+0.85
#6	2.5	4	1	1.14	0.5	3	2	1.65	+0.51	0.5	3	2	1.35	+0.21
#5	2	4	2.5	1.25	1	4	2	1.22	+0.03	1	3	2	1.45	+0.20

* *ΔGT = follow‐up − baseline; positive values indicate gain and negative values indicate reduction. Recession depth (RD), recession width (RW), keratinized tissue height (KTH), and gingival thickness (GT), with the corresponding gingival thickness gain (ΔGT). All values are expressed in millimeters (mm).

### Surgical approach

Surgery was performed by a single experienced periodontist (Marilia Bianchini Lemos Reis),^31^ following the extended CAF technique with partial‐thickness approach as described by Barros et al.^13^ After intraoral antisepsis with 0.12% chlorhexidine digluconate rinse for 30 s, local anesthetic (4% articaine with 1:100,000 epinephrine).† Two vertical releasing incisions were placed at the mesial line angle of tooth #7 and the distal line angle of tooth #3 to allow wide flap mobilization while maintaining a safe distance from the recession margins (Figure 1B). Sulcular incisions were connected to the vertical incisions, including the interdental papillae within the flap design. Partial‐thickness dissection was carefully performed using a 15C blade,‡ parallel to the mucosal surface and extending beyond the mucogingival junction to achieve passive, tension‐free coronal advancement (Figure 1C). Exposed root surfaces were debrided using manual curettes and conditioned with 24% EDTA^32^ gel for 2 min, followed by saline irrigation. The PADM§ was rehydrated in sterile saline, trimmed to fit the defective area, and positioned 1 mm apical to the CEJ area^13^ (Figure 1D). The matrix was stabilized to the periosteum with resorbable 6‐0 Vicryl sutures.** The flap was then advanced to a position 1 mm coronal to the CEJ, fully covering the matrix (Figure 1E), and sutured without tension using a combination of sling and interrupted sutures with 5‐0 blue ultrasoft polyamide thread.†† No periodontal dressing was applied.

### Postoperative protocol

The patient was prescribed 500 mg of amoxicillin every 8 h for 7 days. Ibuprofen 400 mg was recommended every 6 h as needed for pain, for up to 3 days. Oral hygiene instructions included avoiding brushing at the surgical site for 2 weeks, adhering to a soft diet, and rinsing twice daily with 0.12% chlorhexidine digluconate for 14 days. Sutures were removed after 14 days. The patient attended regular maintenance visits for prophylaxis and clinical monitoring.

### Digital assessment of soft tissue

**FIGURE 2:**
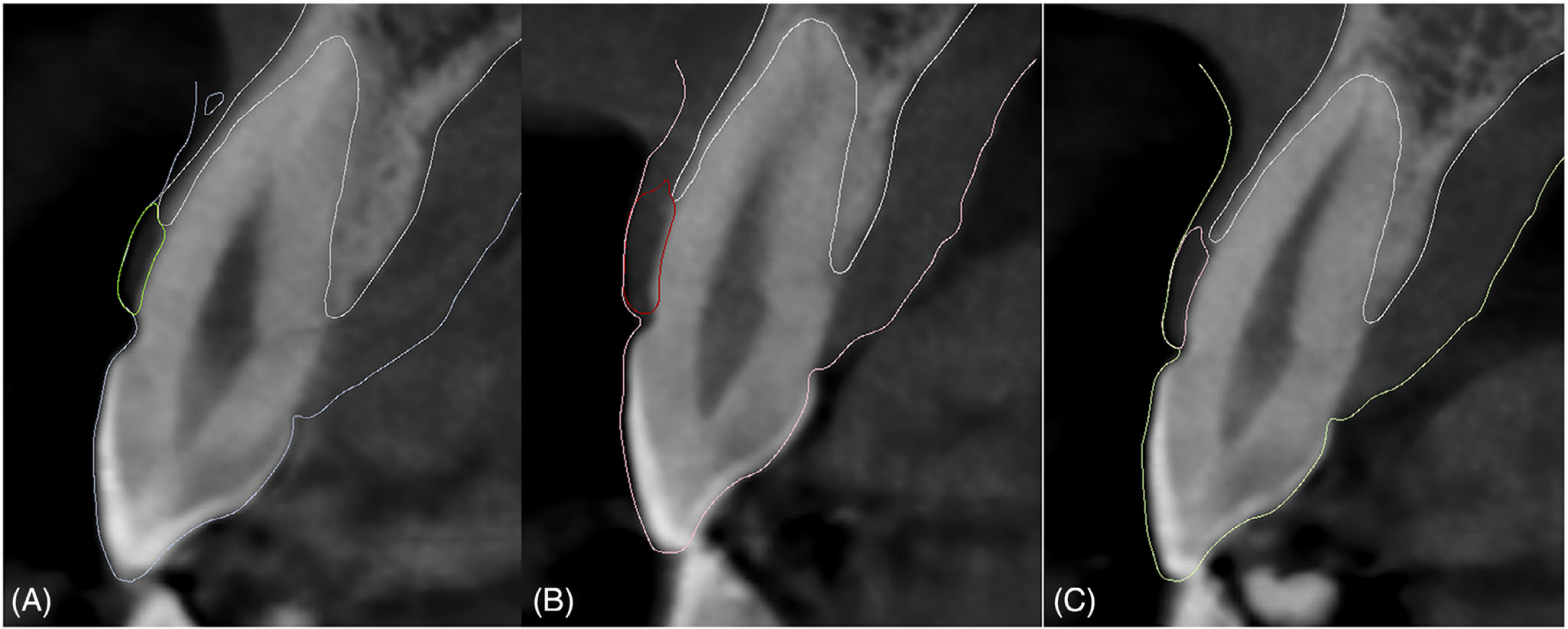
Representative single‐tooth example (#6). Segmented soft‐tissue contours at (A) baseline (green), (B) 6 months (red), and (C) 12 months (pink). Co‐registered STL–CBCT datasets were used to segment the buccal soft tissue; colored outlines indicate the segmented masks at each time point. This figure illustrates segmentation only; gingival thickness (GT) was measured at the alveolar crest (AC) level at the mid‐buccal point as the shortest perpendicular distance from the mucosal surface to the AC.

Intraoral scans and CBCT were performed at baseline, 6 months, and 12 months. All CBCTs used small FOV, low‐dose protocols in adherence to ALARA. Bone segmentation was performed on CBCT using AI‐assisted‡‡ software,^32^ followed by manual refinement to correct for potential underestimation and improve anatomical accuracy.^14^, ^25^ GT measurements at the alveolar crest were taken with reference to superimposed STL files.§§ Figure S1 depicts the measurement scheme.^14^, ^25^ STL models were superimposed and co‐registered to CBCT volumes*** to guide soft‐tissue segmentation of the region from #7 to #3. Segmented meshes were used for volumetric quantification of buccal soft tissue changes over time (Figure 2).

### Patient‐reported outcomes

Patient satisfaction was assessed using a visual analog scale (VAS) at baseline, 6 months, and 12 months. The VAS ranged from 0 (no pain/discomfort) to 10 (worst possible pain/discomfort).

## RESULTS

Clinical evaluations at baseline, 6 months, and 12 months are presented in Table 1 and illustrated in Figure 1. Complete root coverage was achieved at tooth #7, with partial coverage at teeth #6 and #5. Tooth #7 (non‐grafted site) showed improvement in GT, increasing from 0.80 mm at baseline to 1.85 and 1.65 mm at 6 and 12 months, respectively. At teeth #6 and #5, keratinized tissue thickness changed from 1.14 and 1.25 mm at baseline to 1.65 and 1.22 mm at 6 months, and to 1.35 and 1.45 mm at 12 months, respectively (Table 1).

**FIGURE 3:**
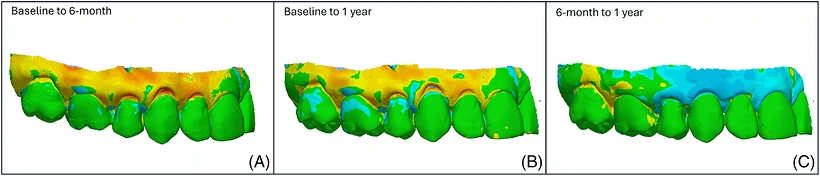
Three‐dimensional evaluation of the buccal soft tissue of five maxillary teeth (teeth #7–#3) using STL mesh superimposition revealed an initial volume gain followed by partial remodeling. (A) Baseline – 6 months, (B) Baseline – 12 months, (C) 6 months – 12 months. Distance maps indicated areas of expansion, contraction, and tissue stability over the follow‐up period.

CBCT measurements at the alveolar crest confirmed GT increases over time (Table 2). Volumetric analysis (Figure 3) demonstrated buccal soft‐tissue volume increasing from 329.70 mm^3^ at baseline to 454.81 mm^3^ at 6 months, decreasing to 345.25 mm^3^ at 12 months This corresponds to a net gain of +15.55 mm^3^ from baseline to 12 months (+4.72% vs baseline) and a reduction of −24.09% relative to the 6‐month peak (+37.95% vs baseline at 6 months).

**TABLE 2:** Cone‐beam computed tomography (CBCT)‐derived gingival thickness (GT) at the alveolar crest measured at baseline, 6 months, and 12 months.

Tooth	Baseline (mm)	6 months (mm)	Gain (6 months − baseline) (mm)^*^	12 months (mm)	Gain (12 months − baseline) (mm)^*^
#7	1.03	1.53	+0.50	1.51	+0.48
#6	1.20	1.55	+0.35	1.64	+0.44
#5	1.37	1.81	+0.44	1.68	+0.31

* Gain = follow‐up − baseline; positive values indicate gain and negative values indicate reduction.

Color‐coded deviation maps indicated volume gain (red/orange) in the cervical third at both follow‐ups, with some localized reduction (blue) between 6 and 12 months. Overall, contour stability was maintained at 12 months (Figure 3). VAS scores for pain/discomfort decreased from baseline to 6 months and remained low at 12 months (Table 3).

**TABLE 3:** Visual analog scale (VAS) scores for tooth‐specific pain/discomfort at baseline, 6 months, and 12 months.

Tooth	Baseline (VAS)	6 months (VAS)	12 months (VAS)
#7	4	0	0
#6	5	0	2
#5	4	3	3

VAS: 0 = no pain/discomfort; 10 = worst possible.

## DISCUSSION

This case demonstrates that mucogingival surgery can achieve precise and predictable root coverage outcomes. In this patient, the use of a PADM promoted increased GT and maintained a stable buccal contour for up to 12 months, aligning with previous reports that support soft‐tissue substitutes as reliable, long‐term alternatives to CTG,^11^, ^13^ particularly for the treatment of multiple adjacent recessions while avoiding donor‐site morbidity.^11^, ^13^ The progressive improvement in patient‐reported satisfaction reinforces the clinical relevance of this approach.

A notable aspect of this case is the methodology applied for soft‐tissue evaluation, which represents a highly accurate protocol used for the first time in this clinical context. By integrating clinical examination, CBCT, and 3D registration of intraoral scans, the approach enabled precise and reproducible volumetric quantification of soft‐tissue changes. Color‐coded deviation maps provided a visual and volumetric representation of tissue gain and remodeling over time, capturing subtle changes that conventional two‐dimensional metrics might miss. Combining STL superimposition with CBCT ensured consistent registration across time points, improved correlation between measured changes and specific regions of interest, and minimized potential overestimation from linear measurements alone.^14^, ^25^ CBCT‐based GT measurements have shown close agreement with clinical methods, and comparative studies against histology (gold standard) have confirmed their accuracy.^14^, ^17^, ^25^

Three‐dimensional workflows also offer a near‐realistic understanding of soft‐tissue morphology and healing dynamics. Superimposition of serial STL models has demonstrated volumetric gains with both CTG and PADM.^16^, ^23^, ^24^, ^26^, ^32^, ^33^, ^34^, ^35^, ^36^ Implementing STL‐based workflows has shown that soft‐tissue healing dynamics can be tracked at 3‐ and 6‐month intervals in root coverage procedures using CTG and mucosal substitutes, including longer‐term follow‐ups of up to five years.^16^, ^23^, ^24^, ^26^, ^27^, ^33^, ^34^, ^35^, ^36^, ^37^ Most studies perform volumetric measurements by comparing baseline STL models, overlaid on the recession area, to determine the volume gained. However, the initial volumetric value is not always known, which may limit interpretation. Segmentation studies in guided bone regeneration have demonstrated the use of CBCT‐based segmentation for hard‐tissue evaluation.^20^, ^21^ Unlike bone, our focus is soft‐tissue analysis, which presents distinct challenges due to morphological variability; using STLs as reference points allows precise segmentation of regions of interest, yielding a more realistic understanding of the healing process.

From a clinical perspective, PADM offers a practical and minimally invasive alternative for treating multiple GRs. In this case, patient‐reported pain/discomfort on the VAS decreased over time and remained low at 12 months. Nonetheless, limitations include the single‐case design, lack of a control group, possible influence of CBCT resolution and operator‐dependent segmentation, and follow‐up limited to 12 months. Future research should involve larger cohorts, extended observation, and artificial intelligence‐assisted segmentation to enhance precision and reproducibility.

## CONCLUSION

PADM‐assisted root coverage resulted in increased GT, stable buccal contour, and high patient satisfaction at 12 months. The integration of intraoral scanning, CBCT, and digital segmentation enabled precise measurements and objective follow‐up, underscoring the clinical relevance of digital technologies in periodontics.

## AUTHOR CONTRIBUTIONS

*Conceptualization*: Edgar Daniel Vargas‐Quiroga, Bruna S.H. Tonin, and Marilia Bianchini Lemos Reis. *Methodology*: Edgar Daniel Vargas‐Quiroga, Bruna S.H. Tonin, Marilia Bianchini Lemos Reis, and Giovana Fernanda Favero da Silva. *Data Curation*: Edgar Daniel Vargas‐Quiroga. *Writing—Original Draft*: Edgar Daniel Vargas‐Quiroga, Bruna S.H. Tonin, and Marilia Bianchini Lemos Reis. *Writing—Review & Editing*: Edgar Daniel Vargas‐Quiroga, Bruna S.H. Tonin, and Marilia Bianchini Lemos Reis. *Supervision*: Arthur B. Novaes Jr. and Marilia Bianchini Lemos Reis.

## CONFLICT OF INTEREST STATEMENT

The authors declare no conflicts of interest.

## Supporting information



Supporting Information

## Footnotes

* UNC‐15; Hu‐Friedy, Chicago, IL, USA.

† DFL, Rio de Janeiro, RJ, Brazil.

‡ Swann‐Morton, Sheffield, UK.

§ NovoMatrix RTM;BioHorizons, Birmingham, AL, USA.

** Ethicon, São José dos Campos, SP, Brazil.

†† Techsuture, Bauru, SP, Brazil.

‡‡ BlueSkyPlan; BlueSkyBio, Grayslake, IL, USA.

§§ Primescan; Dentsply Sirona, Bensheim, Germany.

*** Geomagic 3D Systems, Rock Hill, SC, USA.
