# Supplementary material for: Volumetric analysis of root coverage with porcine acellular dermal matrix: Case study
Source: Clin Adv Periodontics. 2026 Feb 19;16(3):562–9. doi: 10.1002/cap.70029 (PMC13603054; doi:10.1002/cap.70029)
Supplement: Supplementary file 1 [file CAP-16-562-s001.docx]

**Supplementary material**

Root Coverage With Porcine Acellular Dermal Matrix: A Case Report

**Authors:**

Edgar Daniel Vargas-Quiroga, DDS, MSc¹; Giovanna Fernanda Favero da Silva, DDS, MSc¹; Bruna S. H. Tonin, DDS, MSc, PhD²; Arthur Belém Novaes-Jr, DDS, MSc, PhD¹; Marilia Bianchini Lemos Reis, DDS, MSc, PhD¹

**Affiliations**

¹ Department of Oral & Maxillofacial Surgery and Periodontology, Ribeirão Preto School of Dentistry (FORP), University of São Paulo, Ribeirão Preto, SP, Brazil

² Department of Restorative Dentistry, Ribeirão Preto School of Dentistry (FORP), University of São Paulo, Ribeirão Preto, SP, Brazil

***Supplementary Figure S1.*** CBCT-based gingival thickness (GT) measurement at the alveolar crest (AC) level at the mid-buccal point for the treated teeth (#7, #6, #5) at baseline, 6 months, and 12 months. The caliper indicates the shortest perpendicular distance from the external mucosal surface to the most coronal point of the AC. Values correspond to those reported in Table 2.
